# Catching babies, carrying traditions: the voices and practices of traditional birth attendants in Mayuge District, East central Uganda

**DOI:** 10.1186/s12978-025-02251-3

**Published:** 2026-01-10

**Authors:** Enid Kawala Kagoya, Proscovia Auma, Mugabi Joshua, Elizabeth Kawala, Deogratias Asabawebwa, Richard Mugahi, Brenda Doreen Mutunda, Richard Gamubaka, Agnes Namaganda, Allan G. Nsubuga, Jackline Akello, Paul Waako, Kenneth Mugabe

**Affiliations:** 1 Institute of Public Health, Department of Community Health, Busitema University, faculty of health sciences, P.O Box 1460 , Mbale, Uganda; 2 Department of Obstetrics and Gynecology, Busitema University, faculty of health sciences, P.O Box 1460, Mbale, Uganda; 3 Mayuge District Local Government, P.O Box 1317, Mayuge, Uganda; 4 Department of Reproductive, Maternal and Child Health, Ministry of Health, P.O Box, 7272, Kampala, Uganda; 5 Department of Physiology, Makerere University, College of Health Sciences, P.O Box 7062, Kampala, Uganda; 6 Kitibwa Restoring Hope Foundation, Bredford, P.O Box 1025, Massachusetts, United States of America; 7 Department of Obstetrics and Gynecology, Makerere University, College of Health Sciences, P.O Box 7062, Kampala, Uganda; 8 Department of Pharmacology and Therapeutics, Busitema University, faculty of health sciences, P.O Box 1460 , Mbale, Uganda; 9 Clinical Epidemiology Unit, Makerere University, College of Health Sciences, P.O Box 7062, Kampala, Uganda

**Keywords:** Traditional birth attendants, Maternal and newborn health, Socio-cultural determinants, Referral practices, Health system integration, Rural uganda

## Abstract

**Background:**

Traditional Birth Attendants (TBAs) continue to support maternal and newborn care in rural Uganda, particularly where access to formal health facilities is limited. Their roles are deeply embedded in local culture and community trust, yet national policies provide inconsistent guidance on their engagement within the formal health system.

**Objective:**

To explore the evolution, experiences, practices, challenges, and perceived support needs of TBAs in Mayuge District, and examine their interaction with formal health structures.

**Methods:**

A qualitative narrative study was conducted with 15 purposively selected TBAs from all sub-counties of Mayuge District. In-depth interviews lasting 60 to 90 min were held at participants’ homes or workplaces. Participants described and demonstrated their maternal care practices. Audio recordings were transcribed, translated, and analyzed thematically.

**Results:**

Seven key themes emerged from the study: (1) Historical Developments and Evolution of TBAs, highlighting diverse pathways into practice such as apprenticeship, family mentorship, formal Ministry of Health training, and community programs; (2) Practices, Methods, and Approaches, detailing TBAs’ provision of antenatal, delivery, and postnatal care, and management of maternal and newborn complications using both traditional and modern methods including newborn resuscitation; (3) Infection Prevention and Control, revealing variable use of personal protective equipment and inconsistent waste disposal practices constrained by limited resources; (4) Maternal and Perinatal Death Surveillance and Response (MPDSR) Process, illustrating TBAs’ limited participation in formal death notifications and reviews; (5) Referral Process, highlighting complex referral behaviors involving health facilities, fellow TBAs, and traditional healers; (6) Socio-Cultural Influences on TBA Services, encompassing the impact of poverty, gender roles, community trust, and cultural norms; and (7) Challenges in Providing Safe and Effective Care, including barriers such as negative attitudes from health workers, transport difficulties, competition, and lack of formal support.

**Conclusion:**

TBAs in Mayuge District play a vital role in culturally grounded maternal care but face numerous challenges due to limited formal integration and support. Strategies are needed to strengthen collaboration, provide targeted training, and clarify policy to improve maternal and newborn health outcomes in underserved areas.

**Supplementary Information:**

The online version contains supplementary material available at 10.1186/s12978-025-02251-3.

## Introduction

Traditional Birth Attendants (TBAs) remain vital providers of maternal and newborn care in many rural and underserved areas of low- and middle-income countries (LMICs), where access to formal health facilities is often limited [1]. In Uganda, particularly in districts like Mayuge, TBAs frequently serve as the first point of contact for pregnant women, especially in remote communities with inadequate healthcare infrastructure [2]. Their sustained popularity is driven by community trust, cultural acceptance, and practical necessity, despite concerns regarding their limited formal training and reliance on traditional practices [3].

TBAs in Uganda typically acquire skills through apprenticeship, experiential learning, or community-based informal training, with some accessing periodic sessions provided by the Ministry of Health (MOH) or non-governmental organizations (NGOs) [4]. They provide a range of services including antenatal care, delivery assistance, and postpartum support, often managing complications with a mix of traditional remedies and basic biomedical interventions [5]. However, gaps in their knowledge about critical conditions such as eclampsia, obstructed labor, and postpartum hemorrhage contribute to increased risks for mothers and newborns [6].

Despite these challenges, TBAs continue to fill crucial gaps in maternal healthcare delivery, especially where formal services are inaccessible or perceived as unfriendly [7]. Yet, their role within Uganda’s healthcare system remains ambiguous, compounded by policy bans on TBA-assisted deliveries and limited integration into formal health structures [8]. This ambivalence has contributed to systemic neglect, insufficient support, and strained relationships between TBAs and health facilities.

This study therefore aimed to explore the evolution, experiences, practices, challenges, and perceived support needs of TBAs in Mayuge District. It examined their origins, diverse training pathways, and practical approaches to maternal care, as well as the barriers they face and their interactions with formal health systems. By centering the voices of TBAs, the study sought to generate evidence to inform national health policy on the potential for pragmatic collaboration and integration of TBAs within Uganda’s maternal and newborn health framework, ultimately aiming to improve health outcomes in underserved communities.

### Objective

 To explore the evolution, experiences, practices, challenges and perceived needs of TBAs in Mayuge District, and examine how they interact with formal health structures.

### Study site

Mayuge District is located in the southeastern region of Uganda. It is bordered by Lake Victoria to the south, with other neighbouring districts such as Jinja to the northwest, Iganga to the northeast, and Kamuli to the east. The district is part of the Busoga sub-region and it has both rural and urban areas, with Mayuge Town Council as the district headquarters. It has a largely agrarian economy, with most of its population practicing subsistence farming. The population is predominantly rural, with most residents living in villages scattered throughout the district. Rural communities often have limited access to healthcare, education, and other amenities. Due to its rural nature and inadequate formal healthcare facilities, Mayuge District relies heavily on community-based healthcare services, including TBAs, who provide maternal and neonatal care to expectant mothers. Access to skilled medical care, particularly in emergencies is limited and many women in these areas continue to depend on TBAs for assistance during childbirth.

Healthcare infrastructure in the district is underdeveloped, with a shortage of healthcare professionals, including Skilled Birth Attendants (SBAs), and inadequate access to essential maternal and neonatal health services. This has contributed to a high reliance on TBAs, whose services are seen as more accessible and culturally acceptable, despite the risks associated with their lack of formal medical training. Efforts to improve maternal health outcomes in the district are ongoing, with the government and various NGOs working to increase access to formal healthcare services, promote skilled birth attendance, and integrate TBAs into the formal healthcare system.

### Study design

This study employed a narrative qualitative design to explore the experiences, practices, challenges, and support needs of Traditional Birth Attendants (TBAs) in Mayuge District, East Central Uganda. This design was used because it allowed participants to share their personal stories in depth, providing insights into how they understood and navigated their roles within their communities. It was particularly suitable for capturing the rich, contextualized experiences of TBAs, whose work is deeply embedded in cultural traditions, social relationships, and local health-seeking behaviors.

## Materials and methods

We conducted a narrative qualitative study across all sub-counties of Mayuge District, Uganda. Fifteen in-depth interviews were held, with at least one Traditional Birth Attendant (TBA) recruited from each sub-county using snowball sampling techniques. The interviews were conducted at the residences or work premises of the TBAs to ensure comfort and contextually rich engagement. All interviews followed a semi-structured interview guide, were audio-recorded, and accompanied by simultaneous note-taking.

The data collection team consisted of two experienced female qualitative researchers with backgrounds in public health and medical anthropology. They were fluent in the local language (Lusoga), had prior fieldwork experience in maternal health studies, and were trained in qualitative interviewing techniques. Their familiarity with the cultural setting and non-clinical positioning helped build trust with participants and reduce social desirability bias.

We employed a narrative qualitative research design to centre participants’ voices, lived experiences, evolving practices, and the stories they use to make sense of their work and challenges. Reflexive thematic analysis, as outlined by [9], guided the interpretation of both content and structure of narratives. This method allowed us to identify recurring patterns while preserving the integrity of individual accounts. Our analysis was grounded in a constructivist epistemology, aligned with the ‘big Q’ qualitative paradigm that views knowledge as co-constructed between researcher and participant.

Coding was both deductive guided by the study’s objectives and inductive, allowing unanticipated themes to emerge. Data analysis was facilitated using Dedoose software. The research team reviewed and refined the codes collaboratively through iterative discussions, leading to the development of themes and subthemes that captured both expected and emergent insights from the narratives.

### Ethical consideration

Ethical approval was obtained from the Mbale Regional Referral Hospital Research and Ethics Committee (MRRH-2023-342). All procedures were conducted in accordance with the ethical standards of the institutional research committee and the 1964 Helsinki Declaration and its later amendments or comparable ethical standards. Prior to participation, each Traditional Birth Attendant (TBA) was provided with clear and comprehensive information about the purpose, procedures, risks, and benefits of the study in a language they understood. Verbal and written informed consent were obtained before each interview. TBAs were assured of confidentiality, voluntary participation, and the right to withdraw at any time without consequence. Interviews were conducted in private spaces at the TBAs’ residences or work premises to ensure comfort and privacy during data collection.

## Results

### Social demographic factors

Fourteen female and one male TBAs participated in the study (*n* = 15), They averaged 45.2 years of age (median: 44; IQR: 38.5, 52.5) and 14.7 years of experience (median: 12; IQR: 8.5, 18), (see Table 1)

**Table 1:** Shows the demographic characteristics of the participants

Characteristic	Category	*n*
Age group (years)	30–39	5
	40–49	4
	50–59	3
	60 and above	3
Gender	Female	14
	Male	1
Years of experience	5–9 years	5
	10–19 years	5
	20–29 years	2
	30 years and above	3
Location	Bukatuube	2
	Mayuge	2
	Mpungwe	2
	Others (one TBA each)	9

“Others” includes Buguwa, Wangobu, Nawanvubu, Waitambogwe, Buwalima, Kigulu, Buwaiswa, Kikuubo, and Nansaga

### Thematic areas

Seven key themes emerged from the study, capturing the evolution, practices, infection control efforts, MPDSR participation, referral patterns, socio-cultural influences, and challenges faced by Traditional Birth Attendants (TBAs) in Mayuge District (See Table 2).

**Table 2:** Thematic areas from the study

Theme	Subtheme	Subtheme/Child Node	Finding
Historical Developments and Evolution of TBAs	Evolution	Apprenticeship	Learning through hands-on mentoring by experienced TBAs
		Formal Training by MOH	Participating in structured training sessions by the Ministry of Health
		Former VHTs	Transitioning from Village Health Teams to traditional birth practice
		Motivation by Community	Encouraged by local demand and recognition from community
	Training Pathways	Community Training Programs	Informal sessions within the community to share skills
		Training by Civil Society Organizations	Workshops and trainings offered by NGOs and CSOs
	Training Packages	Postnatal Care	Teaching TBAs how to care for mothers and babies after birth
		Antenatal Care	Supporting pregnant women with health advice and checkups
		Delivery and Childbirth	Assisting women during labor and delivery using learned techniques
		Management of Some Conditions	Managing selected maternal and neonatal health issues
		Helping Babies Breathe	Applying newborn resuscitation methods to save non-breathing babies
Practices, Methods and Approaches	Identification and Diagnosis of Conditions	Preeclampsia and Eclampsia	Recognizing symptoms like high blood pressure and seizures
		Birth Asphyxia	Detecting lack of breathing in newborns at birth
		Maternal Sepsis	Identifying signs of infection during or after childbirth
		Postdate (Deeni)	Recognizing pregnancies that go beyond the expected term
		Postpartum Hemorrhage	Noticing excessive bleeding after delivery
		Placenta Previa	Observing bleeding that may indicate low-lying placenta
		Breech Deliveries	Identifying babies presenting buttocks or feet first
		Incomplete Abortions	Recognizing retained fetal tissue after miscarriage
	Conditions Managed by TBAs	Preeclampsia/Eclampsia	Managing through herbs or referrals to health facilities
		Birth Asphyxia	Conducting manual stimulation or mouth-to-mouth resuscitation
		Postdate (Deeni)	Using herbs or techniques to stimulate labor
		Nabuguma (Heat in abdomen)	Using traditional beliefs and herbs for abdominal discomfort
		Abortions	Offering support or traditional means for incomplete abortions
		Neonatal Resuscitation	Using basic resuscitation skills on newborns
		Postpartum	Providing aftercare for mothers including herbal remedies
		APH	Attempting to control bleeding or referring cases urgently
	Medicines Used	Modern Medicines	Administering available over-the-counter or prescribed drugs
		Traditional Medicines	Applying local herbs and remedies based on ancestral knowledge
Infection Prevention and Control	Disposal of Waste	Burial Method	Burying delivery waste in shallow pits
		Placenta Pit	Using specific pits for safe placenta disposal
		Dispose in Garden	Disposing of biodegradable waste in home gardens
		Burning	Locally burning waste materials like gloves and pads
	Personal Protective Equipment	Use of Gloves	Wearing gloves during delivery for protection
		Black Kaveera	Using polythene bags as makeshift protective gear
	Handwashing Practices	Soap Use	Washing hands with soap before handling clients
		Use of Cooking Oil	Using cooking oil when soap is unavailable
MPDSR Process	Death Notifications		Reporting maternal and neonatal deaths in the community
	Perinatal Death Reviews		Reviewing newborn or stillbirth deaths for learning
	Maternal Death Reviews		Evaluating maternal deaths to understand the causes
Referral Process	Referral to Health Facility		Sending complicated cases to formal health facilities
	TBA to TBA Referral		Referring clients to more skilled TBAs
	Referral to Traditional Healers		Recommending spiritual or traditional intervention
Socio-Cultural Influences on TBA Services	Poverty Among Mothers	Inability to Afford Medical Care	Financial hardship limits access to hospitals
		Ignorance About Better Medical Care	Limited knowledge of available services or their benefits
	Husband Influence	Husband Preferences	Husbands dictate birthing location and care options
		Fear of Becoming Widowers	Men fear maternal death from hospital interventions
	Mother-in-law Influence	Cultural Expectations	Pressure to follow traditional childbirth practices
	Motivation by Community	Community Influence	Continued practice due to local trust and reliance
Challenges in Providing Safe and Effective Care	Ban by MOH	Working Under Fear	Practicing in secrecy due to official prohibition
	Contextual Factors	Failure to Disclose HIV Status	Limits TBAs’ ability to take precautions
		Lack of HIV Testing Kits	TBAs are unable to screen clients
		Lack of Transport	Delays in referring emergency cases to hospitals
		Harsh Health Worker Attitudes	Negative reception at health centers discourages referrals
		Lack of Space	No designated area for deliveries and rest
		Unprepared Mothers	Clients arrive late or without necessary supplies
	Competition	TBA vs. TBA	Rivalries among TBAs hinder collaboration
		TBA vs. VHTs	Conflicts with Village Health Teams over roles
		Private Clinics	Clinics attract clients with more resources
	Lack of MOH Support	No Supplies or Recognition	TBAs are excluded from formal systems and lack materials
	Lack of Patient Sundries	Gloves and Kaveera Not Provided	Mothers don’t bring basic supplies needed for delivery
	Patient Negligence	Delays and Poor Treatment Adherence	Mothers arrive late or fail to follow instructions
		Poor ANC Visits	Lack of antenatal care leads to poor outcomes

#### Theme 1: Historical developments and evolution of TBAs

The study found that TBAs evolved through both informal apprenticeship and formal training. Initially, they learned by working with experienced practitioners, later gaining skills through Ministry of Health programs. Many had served as Village Health Teams, giving them a strong foundation in basic care. Community support motivated their work, and training focused on antenatal, postnatal, and emergency childbirth care.

##### Subtheme: Evolution of traditional birth attendants

TBAs acknowledged the changing health landscape and the enduring role of community-based birth services. They passed down skills through informal learning methods, primarily through apprenticeship. Learned from older, more experienced attendants who shared their knowledge through hands-on experience rather than formal education. This apprenticeship model allowed them develop practical skills and knowledge tailored to the specific needs of the communities they served.

##### Subtheme: Apprenticeship

TBAs reported that their initial training came through apprenticeship, where they learned directly from experienced practitioners. This informal training allowed them to gain hands-on knowledge and skills necessary for managing deliveries, despite the lack of formal educational structures.

*“Truthfully*,* it is familial work because my paternal grandmother did the same and some paternal aunts.”(TBA11*,* Female*,* 38 years*,* Mayuge Village)*.

*“My mother was a TBA*,* she trained me and also delivered all of us from TBAs*,* and I too have never produced in the hands of a skilled health worker.”(TBA9*,* 44 years*,* Mpungwe Village)*.

##### Subtheme: Formal training by MOH

Over time, some TBAs received formal training through the Ministry of Health programs. The training enhanced their skills and brought their practices closer to modern medical standards. TBAs highlighted that the formal training significantly improved their ability to handle complications and provided more structured knowledge.

"Now, when the training came up about this, they came and looked for us and took us to Kigandalo, where we were taught what we didn’t know. Like how to examine for the gestation age using the distention of the abdomen, which we now try, but before that, we were working like we used to initially. (TBA10, 32 m years, Bukatuube Village)".

##### Child node: Former village health team

Most TBAs had previously served as VHTs, giving them a foundation in maternal and child health and familiarity with the broader health system. This experience made their transition to TBA work smoother, as they were already engaged in community health and had basic health training that supported their new roles.

*“I am actually a VHT and while at the facility*,* I interact with midwives and observe how they handle these mothers*,* learn and also come this side and handle them using the skills attained.” (TBA11*,* 38 years*,* Mayuge Village)*.

##### Child Node: Motivation by community

The study found that community support strongly motivated TBAs, giving them purpose and reinforcing their role in maternal and child health. As healthcare needs grew, formal MOH training became essential, equipping TBAs with basic medical skills to align with modern standards and reduce maternal and neonatal mortality.

*“Now since that day*,* I stayed with the health worker from nakavule hospital for some time*,* the Hospital had not yet reached where it is right now*,*.*,* this the health worker invited me after she had seen that I can do that work. Then i went to that health worker’s home*,* which was near CMS. She requested me to help keep her home where she used to stay. Whenever she would go to work*,* she sometimes would come back abruptly*,* maybe she wanted to test my ability to assist delivery*,* she would find when I had delivered like three mothers and their babies were fine. So*,* the community urged me to continue.”(TBA*,* 60 Years*,* Kigulu Village)*.

What stood out prominently was the role of community-driven motivation in shaping the evolution of TBAs. Community members supported their local TBAs not merely for their accessibility, but because they were regarded as essential figures within the local healthcare system. The trust placed in them stemmed from their longstanding relationships and deep-rooted connections within the community, often making them the preferred providers of maternal care.

"I started this job as a house help, I come and stay with TBA as a house help for your children but as time goes by the TBA asks me to stay with some of her patients. I observe what the TBA is doing during the delivery, so when they left the patient there with me, the patient asked for where my boss has gone, and before i know it, she had started pushing her child. By then I was still young and had not yet gotten married. I asked the mother what I should do and she told me to go there with a thread and tie the cord 3 times. And I did so. Then she told me to put my fingers in the baby’s mouth and I did so very fast. But for that baby, she produced it while it was lying flat. So, she told me to carry the baby and place it on her belly and I asked her, “Won’t the baby die?” She said no, I just put the baby on her belly. I cleaned her blood until she stopped bleeding.”(TBA14, 53 years, Bukatuube Village)".

##### Subtheme: Training pathways

The study reported that TBAs engaged in community training programs that were often localized to meet the unique needs of their communities. These programs provided TBAs with essential skills that were culturally relevant and practical. Additionally, training by civil society organizations further supported TBAs by offering structured educational opportunities, resources, and guidance, which complemented their informal practices.

##### Child node: Community training programs

TBAs reported that local community-based training programs were instrumental in improving their knowledge and skills. These programs focused on maternal health topics, including antenatal care, safe delivery practices, and postnatal care, which helped bridge gaps in formal education.

*“We have a community group of TBAs and we meet once in a while for a refresher and also orient the new TBAs.” (TBA12*,* Female*,* 39years*,* Nansaga Village)*.

##### Child node: Training by civil society organizations

Civil society organizations played a role in providing training to TBAs. TBAs acknowledged that the civil society organizations contributed to their development by offering specialized courses and resources, especially in rural areas where government support was limited.

*“I can’t remember which organization it was but there were some people from some organization who met with us at Mpuugwe primary school and took us through family planning and other things.” (TBA7*,* Male*,* 46 years*,* Buwaiswa Village)*. 

##### Subtheme: Training by their husbands

Some TBAs acknowledged that they were trained by their husbands, who often passed on the knowledge and skills needed for childbirth assistance within the family or community. This practice of male involvement in training, though not always widely recognized, has been an essential part of the support system in certain cultures. In many cases, these husbands, often experienced midwives themselves, played a crucial role in mentoring their wives, ensuring the continuity of traditional birthing practices.

*“When I came here*,* God tested me that my husband was one of those that used to deliver mothers from the villages this side so he taught me and I now deliver mothers and offer other services.” (TBA6*,*60 years*,*)*.

*“Like 20 years*,* as I told you my husband was initially a TB*,* i used to observe as he worked and he taught me some of the thing*,* when mothers came to our home to deliver*,* I used to also help him when he was away but he didn’t know at first that I used to do so because by then I was still young when mothers came they usually asked for him to come and attend to them..”(TBA4*,* Female*,* 53 years*,* Mpungwe Village)*.

##### Subtheme: Training packages offered to TBAs

TBAs acknowledged to have received training focused on essential maternal and child health services. These packages included education on postnatal care, antenatal care, delivery, and management of specific conditions, and helping babies breathe the latter a critical life-saving skill for newborns facing respiratory distress. TBAs were trained to handle a wide array of situations, making them indispensable in rural and underserved areas.

*“So*,* they told us that when a mother comes when the pregnancy period is still small*,* we check them and then tell them to return after a month. If they come when the pregnancy period is advanced*,* we also tell them to return because they can come just to see whether everything is fine in the womb and when you examine her you see that everything is okay then she goes but after a short while she returns with pains.”(TBA5*,* Female*,* 54 years*,* Buwalima Village)*.

##### Child node: Postnatal care

TBAs reported learning essential postnatal care practices, including monitoring maternal health after delivery and recognizing signs of complications. This training was critical in reducing maternal morbidity by providing timely intervention during the postnatal period.

*“So*,* we were taught that there are about 7 signs that we note when a mother comes sometime after giving birth. Swelling of the legs*,* or face*,* or even a mother who is not in a good condition. You send them to the facility. The legs of a sick person can be seen*,* mostly shiny but if you press on them the skin delays to return.”(TBA6*,* 60 years*,* Kigulu Village)*.

##### Child node: Antenatal Care

TBAs highlighted that their training in antenatal care focused on identifying early signs of pregnancy-related complications, counseling mothers on proper nutrition, and encouraging health-seeking behaviors. This proactive care helped to improve pregnancy outcomes, especially in rural areas with limited access to formal health services.

*“Really what they told us is what I do. She comes with gloves and a polyethene sheet. The polyethene sheet is placed on the bed where she is going to lay while I use the gloves to examine her. Even when I am examining the abdomen alone*,* I put on gloves. So*,* if I need to examine the other parts*,* she carries extra pairs of gloves.”(TBA4*,* Female*,* 38 years*,* Baitambogwe Village)*.

##### Child node: Delivery and childbirth

TBAs shared that the training provided them with essential techniques for managing deliveries, though their ability to handle complicated cases remained limited without access to medical facilities.

*“Now the mother comes with her pregnancy and requests for a checkup and I check her while putting on my gloves. Because God helped me that I remember what I was taught and still see it as if I first saw it yesterday. Now you can see the baby in the womb and how it is lying and if possible*,* I deliver her from here.” (TBA8*,* Female*,* 39 years*,* Kikuubo Village)*.

##### Child node: Management of some conditions

TBAs also received training in managing specific maternal and neonatal conditions, including complications like preeclampsia, hemorrhage, and neonatal resuscitation. They emphasized that while the training improved their capacity to manage these issues, referral to health facilities was often necessary for severe cases.

*“And if someone has problems in their pregnancy*,* we direct them in the traditional way and God willing the problem is cured. People come and say that they have wounds in their private parts when they are pregnant*,* we know that is syphilis so we manage it and they heal.”(TBA3*,* Female*,* 52 years*,* Nawanvubu Village)*.

##### Child node: Helping babies breathe

TBAs mentioned that specialized training in neonatal resuscitation, specifically in helping babies breathe after birth, was offered to them to helpreduce neonatal mortality. The training gave them the confidence to intervene in cases of asphyxia, although resources for such interventions were often limited.

*“They taught us how to deal with babies*,* especially newborn babies born when they are tired and can’t breathe.” (TBA9*,* Female*,* 44years*,* Mpungwe Village)*.

#### Theme: Practices, methods, and approaches used in the management of obstetric conditions

The study revealed that TBAs used a combination of traditional knowledge and modern medical practices to identify and manage obstetric conditions such as preeclampsia, birth asphyxia, and postpartum hemorrhage. They relied on observation, practical interventions, and referrals when needed. TBAs also integrated both modern medicines and traditional remedies, making them vital providers in areas with limited access to formal healthcare.

##### Subtheµe: Identification and diagnosis of conditions

TBAs identified and diagnosed conditions such as preeclampsia, birth asphyxia, and postpartum hemorrhage based on physical symptoms. They reported using traditional knowledge, such as observing swelling or hypertension but noted that advanced diagnostic tools were often unavailable.

*“People come and say that they have wounds in their private parts when they are pregnant*,* we know that is syphilis. They tell you that “endira” is paining.”(TBA 5*,*Female*,* 54 years*,* Buwalima Village)*.

##### How do you usually manage syphilis?

*“I use herbs. I know them but not by name. I know that this herb and that one when mixed and cooked*,* they can treat this condition.”(TBA 5*,* Female*,* 54 years*,* Buwalima Village)*.

But how do they look like.

*“They are made of wood or husks.”(TBA 5*,* Female*,* 54 years*,* Buwalima Village)*.

Husks like for sweet potatoes?

No husks of trees mostly those we have around this village.”(TBA 5, Female, 54 years, Buwalima Village).

Now how do you distinguish between the ones you require for a certain treatment?

*“I base on what I was told by the people who introduced me to the practice. If I was told that this one is used for this then I know it for that use.So*,* when it is the first time for the mother to come here*,* I take my time to examine her well and know the baby’s status. This is what I use (shows a fetoscope) to know according to the fetal lie and orientation*,* even the stage the baby is at I know it is for delivering out. Even the way she is having her pains you know it’s time.”(TBA 5*,* female*,* 54 years*,* Buwalima Village)*.

##### Child node: Preeclampsia

Preeclampsia was one of the conditions TBAs managed, though they often lacked the tools for proper diagnosis. They identified it through signs like convulsions and swelling, relying on experience and community knowledge. Without equipment such as blood pressure cuffs or urinalysis kits, they provided basic care and referred severe cases to health facilities. TBAs were often the first to respond but expressed concern that limited training made it hard to detect the condition early and prevent serious complications like eclampsia or stroke.

*“We treat those here using traditional medicine. We use paraffin mixed with tea leaves and dust and we scrub her and then cover her and then cover her with a mat.”(TBA10*,* Female*,* 32 years*,* Bukatuube Village)*.

Do you cover her once or cover in intervals?

*“I cover her and when I see she is still shivering I cover her again with more beddings. Now for me I usually have my Panadol in the house so if she is still shivering*,* I give her some tea and some 2 Panadol tablets which in most cases helps to relieve the shivering. I don’t know how it works but it works for me.” (TBA10*,* Female*,*32 years*,* BukatuubeVillage)*.

##### Child node: Birth asphyxia

Birth asphyxia was identified by TBAs through signs such as a lack of breathing or a weak cry immediately after birth. They applied basic resuscitation techniques learned through training, including chest rubbing and mouth-to-mouth resuscitation. However, their ability to manage severe cases was limited by a lack of essential equipment like neonatal resuscitators and oxygen. This compromised the effectiveness of their interventions. Despite these challenges, TBAs emphasized their role in providing immediate care and referring affected newborns to health facilities for advanced treatment

"When mothers come when they are tired, I first give them first aid of a cup of cold hot then I relax them on the bed and wait for the outcome. (TBA15, Female,62 years, Mayuge Village").

So, what happens after?

*“If all goes well*,* they give birth well and we thank God but most times if they fail*,* I call a boda boda and they take them to Iganga.” (TBA15*,* Female*,*62 years*,* MayugeVillage)*.

##### Child node: Maternal sepsis

Maternal sepsis was another condition TBAs attempted to manage, especially after childbirth. They recognized early signs like fever, foul-smelling discharge, and abdominal pain, often using traditional remedies, though these were not always effective. TBAs encouraged women to seek further care if symptoms worsened, but many lacked full understanding of sepsis severity and the need for timely antibiotics. In severe cases, some referred women to health facilities, though delays in transport and patient reluctance were common. Limited medical knowledge and lack of antibiotics hindered effective management, increasing the risk of poor outcomes.

"We use certain herbs and plants that have antimicrobial properties. For example, plant-based concoctions or teas made from roots, leaves, or bark to fight infections and reduce inflammation. (TBA9, Female, 44 years, Mpungwe Village)".

"Warm compressions or poultices made from locally sourced herbs or other substances are applied to the abdomen or other affected areas to soothe pain and potentially promote healing.” (TBA9, Female, 44 years, Mpungwe Village).

##### Child node: Postdate (Deeni)

During the study, TBAs reported frequently encountering postdate pregnancies, known as Deeni. They monitored labor signs and advised women to prepare for delivery after the expected date. Some used traditional methods like herbal teas or abdominal massage to hasten labor but understood the risks, such as stillbirth. TBAs often referred postdate cases to health facilities for monitoring and possible induction, acknowledging that advanced care was needed. Their knowledge helped them recognise the condition early and encourage timely medical attention.

*“We use some herbs like* ***Enfwoddo*** *which relax the uterus and are used to stimulate contractions or prepare the body for labor.”(TBA14*,* Female*,* 53 years*,* Mpungwe Village)*.

*“Emotional and spiritual guidance is offered to reduce stress and encourage relaxation*,* with the belief that a calm*,* balanced state can help trigger labor.” (TBA9*,* Female*,* 44 years*,* Mpungwe Village)*.

##### Child node: Postpartum hemorrhage

Postpartum hemorrhage (PPH) was a common condition TBAs encountered, identified mainly by excessive bleeding after childbirth. TBAs knew from experience that heavy bleeding was life-threatening and required prompt action. They encouraged breastfeeding to promote uterine contraction and used traditional herbal remedies to control bleeding. However, many lacked access to essential medical treatments like uterotonics or blood transfusions. For severe PPH, TBAs referred women to health facilities. The study showed that while TBAs managed mild cases, limited resources and training hindered their ability to handle severe cases effectively.

*“I have been lucky all my years of practice. Allah has so far helped me I have never had such but if she is done giving birth*,* I usually tell her that if there’s any problem*,* she should tell me because I will not check you like I am checking a young person. So*,* she should tell me so that we see what could be wrong.” (TBA9*,* Female*,* 44years*,* Mpungwe Village)*.

##### Subtheme: Conditions managed by TBA and how they are managed

The study revealed that TBAs also recognize and manage other conditions such as preeclampsia, birth asphyxia, postdate pregnancies, and neonatal resuscitation. They used a combination of observation, traditional remedies, and modern medical interventions, although more complicated cases required referrals to health facilities.

##### Child node: Preeclampsia/Eclampsia

The study found that TBAs managed preeclampsia and eclampsia by observing symptoms and using traditional remedies. While they recognized signs like high blood pressure and swelling, they lacked the training and resources to handle severe complications. TBAs often referred affected women to health facilities for medical care but sometimes used ineffective traditional methods, such as herbal remedies or paraffin mixed with water and sugar, highlighting gaps in their training and support.

*“Now while managing amakilo*,* I use those potato leaves. When I tell you this you might despise the leaves but they are medicinal.I know and i have heard of the potential of these potato leaves. You pour water mixed with the extract from the potato leaves on her head if she is shivering because there are 2 types of “amakilo “*,* one is whene the girl doesn’t bathewith the local herbs during pregnancy. For which you just give her the leaves only without even anything else.”(TBA9*,* Female*,* 44 years*,* Mpungwe Village)*.

So how does she use them?

*“She squeezes them in a basin of water and uses them to wash her head.” (TBA9*,* Female*,* 44 years*,* Mpungwe Village)*.

For how long and how many times?

*“Even if it’s just that one time.” (TBA9*,* Female*,* 44 years*,* Mpungwe Village)*.

##### Child node: Birth asphyxia

The study revealed that TBAs often encounter birth asphyxia, where the baby lacks oxygen during delivery. They manage it using traditional neonatal resuscitation methods like clearing airways, stimulating the infant, and positioning to aid breathing. However, their efforts are limited by the lack of advanced tools like oxygen or ventilators. For severe cases, TBAs refer babies to health facilities, but transportation challenges and delays often hinder timely care and successful outcomes.

"Now for me, I think these two people getting tired is due to you as the birth attendant if she is not just sick. As you tell her to push on every contraction, it makes both the mother and the baby tired. It requires you to instruct her to push when you are sure the baby is ready and within a few pushes 4, 5 pushes the baby is out but if you put her through those things of pushing over and over, you even leave her there and first do other things, you might come back when she is tired and she might even rapture.”(TBA9, Female, 44 years, Mpungwe Village)".

Now how do you gauge the sufficiency of the contraction so as to prevent the mother from getting tired?

*“if she comes while in labor*,* I ask for gloves*,* I examine her and tell her that her baby is still far so I tell her to wait to push.” (TBA9*,* Female*,* 44 years*,* Mpungwe Village)*.

What I want to know is how you count those steps (dilations).

"You might check and note that the head is still up or you might tell her to push because you think the baby is near but in actual sense, she is still far. So, you check for the descent of the head and then you tell them to first restif it is still up. After some time, you notice the head has reached and then tell her to push and after a few pushes the baby is out (TBA9, Female, 44 years, Mpungwe Village)".

*“Also*,* for those you give her the leaflets and a little paraffin. She uses it to wash her head and she gets relieved.” (TBA9*,*Female*,* 44 years*,* Mpungwe Village)*.

So, you use the sweet potato leaflets.

*Yes*

*“You drop in some little paraffin and that problem is relieved and if it persists and it seems that you won’t manage*,* you send her to the hospital.” (TBA9*,*Female*,* 44 years*,* Mpungwe Village)*.

##### Child node: Postdate (Deeni)

TBAs diagnosed postdate pregnancies mainly through maternal reports and signs like large belly size or missed labor. They encouraged patience and used traditional methods such as herbal teas or dietary advice to ease labor. Some attempted manual interventions, which were not always effective and sometimes risky. The study found postdate pregnancy was the most common reason mothers sought TBAs, who managed it well, viewing it as an abnormal condition linked to cultural and social factors.

*“We engage them in some cultural practices where they bring a tied elephant grass and “enkaata”*,* so during that ceremony*,* they untie the elephant grass and put the “Enkaata” on their head*,* we draw a line and they jump it*,* that is the process of untying a pregnancy*,* after that event*,* they get contractions.” (TBA15*,* Female*,* 62 years*,* Mayuge Village)*.

Is that all?

*“They come here*,* I mix for them some water and herbs then they bathe for a week*,* they normally don’t take long without getting contractions.” (TBA9*,* Female*,* 44 years*,* Mpungwe Village)*.

##### Child node: Nabuguma (Heat in the uterus)

Nabuguma, or “heat in the uterus,” was commonly managed by TBAs during pregnancy and childbirth. They believed it resulted from excessive internal heat due to stress or strain and treated it with herbal remedies, warm compresses, or abdominal massages. While these methods were thought to relieve discomfort, there was limited evidence of their effectiveness. TBAs relied on traditional knowledge but lacked diagnostic tools to identify if symptoms stemmed from more serious medical issues like infections or pregnancy complications.

*“If you’re losing a pregnancy at around 3 months or 4*,* or you’ve lost like 5*,* I give you those herbs and you take them and we see what we can do*,* the pregnancy grows until you can give birth.” (TBA15*,* Female*,* 62 years*,* Mayuge Village)*.

How many times does one take the herbs in a day?

*“Twice only in a day.” (TBA15*,* Female*,* 62 years*,* Mayuge Village)*.

For how long?

*“For about a month only.” (TBA15*,* Female*,* 62 years*,* Mayuge Village)*.

After that month do they feel better? Does the pregnancy grow well?

*“Yes ma’am” (TBA15*,* Female*,* 62 years*,* Mayuge Village)*.

##### Child node: Abortions

The study found that TBAs often managed spontaneous or induced abortions, especially in rural areas with limited family planning services. For spontaneous abortions, they provided emotional support and helped with recovery, sometimes using herbal remedies for bleeding or pain. In some cases, TBAs were also involved in assisting women seeking induced abortions.

If you’re losing a pregnancy at around 3 months or 4, or you’ve lost like 5, I give you those herbs and you take them and we see what we can do, the pregnancy grows until you can give birth.”(TBA15, Female, 62 years, Mayuge Village).

##### Child node: Neonatal resuscitation

The study found that many TBAs were trained in basic neonatal resuscitation techniques, such as clearing the infant’s airways, stimulating the baby to breathe, and positioning the baby correctly after delivery. However, these methods were often rudimentary, and TBAs lacked the equipment and expertise to perform more advanced resuscitation techniques, such as the use of oxygen or mechanical ventilation. TBAs reported that their success with neonatal resuscitation depended heavily on the timing of the intervention and the severity of the asphyxia.

*“If God allows and the baby comes out well*,* I get her out and hurry up to cut the cord then hold her by the legs and buttocks putting her upside down*,* and take some time while still holding her and you notice she starts breathing. When she breathes for the second time you know you are good. Usually*,* I even first leave the mother and tell her to cover herself so that she doesn’t become cold as I focus on the baby. The baby can have like 2–3 breaths and she starts crying*,* then you know that life is back. Then you cover her very well and you put her aside and then go back to the mother and remove the remaining things.” (TBA15*,* Female*,* 62 years*,* Mayuge Village)*.

So does putting the baby upside down help withbreathing

Yes.

What of squeezing the buttocks?

*“It helps the baby not to let air pass through but rather to remain inside the body because if the baby breathe once the air may passthrough the anal opening and she dies.” (TBA15*,* Female*,* 62 years*,* Mayuge Village)*.

Do you assess the baby’s breathing immediately after birth for example at 1 and 5 min?

*“No for us we are usually doing this and that because we didn’t learn them very well. But also*,* you might be holding the baby and she slips and falls that is why we only try putting her upside down.” (TBA15*,* Female*,* 62 years*,* Mayuge Village)*.

What do you do if a mother gives birth to an exhausted baby from here?

*“You it upside down and beat the legs.” (TBA15*,* Female*,* 62 years*,* Mayuge Village)*.

Like how this one turns the legs to the other side?

*“No*,* you put the legs up and you hit gently on the legs like this and it breathes.”(TBA15*,* Female*,* 62 years*,* Mayuge Village)*.

For how long.

*“Utmost 20minutes.” (TBA15*,* Female*,* 62 years*,* Mayuge Village)*.

Have you ever done it and it refused to work?

*“No*,* it has never failed.” (TBA15*,* Female*,* 62 years*,* Mayuge Village)*.

##### Child node: Prenatal care

The study also reported that TBAs played a role in providing prenatal care to expectant mothers in Mayuge district. They are trusted by many due to their cultural familiarity and experience in childbirth, they also offer postnatal care at their sites and even extend care to the homes of the mothers, and this was evidenced by the presence of postnatal units at their places of work.

"As long as she is still here with me, she can take tea like 2 to 3 times because sometimes she might be in labor but at the sometimes yawning and clearly see that she is hungry. So, I give her a cup of tea with something to accompany the tea and when I see her situation is not becoming better I send them to the Health Centre. And for me nowadays I no longer keep people here for long maybe if itis late at night without transportation.” (TBA15, Female, 62 years, Mayuge Village).

*“I use my hands to feel and know the baby’s lie and you can know the normal and abnormal lie. Even when she comes and doesn’t know the period of pregnancy*,* you can know and tell her that she will give birth in a certain month.”(TBA9*,* Female*,* 44years*,* Mpungwe Village)*.

##### Child node: Postpartum care

The study found that TBAs offered postpartum care, monitoring mothers for complications like PPH, infection, and uterine involution. They provided counseling and physical care, including abdominal massage to help uterine contraction and placental expulsion, and supported breastfeeding. For complications, TBAs often used herbal concoctions to control bleeding, though these methods were not always effective.

*“Rituals*,* prayers*,* or ceremonies are performed to bless the new mother*,* protect her from negative energy*,* and encourage a smooth transition into motherhood. Family members and community support play a key role in the healing process.”(TBA12*,* Female*,* 39 years*,* Nansaga Village)*.

*“After childbirth*,* herbal baths made from leaves*,* roots*,* or flowers are commonly used to cleanse and heal the body. Herbs like rosemary*, *ginger*,* etc. aid in wound healing*,* improve circulation*,* and help with relaxation.”(TBA9*,* Female*,* 44 years*,* Mpungwe Village)*.

##### Child node: Delayed cord clamping

Most TBAs allowed the umbilical cord to remain intact until it naturally stopped pulsating. They believed that waiting for the pulse to cease allowed the baby to receive the full benefits of the remaining blood in the placenta. Others said they waited for the cord to “detach” naturally, either through gravity or when the baby had moved away from the mother, before cutting it, as it symbolized the bond between mother and child and gave the baby time to adjust to the outside world.

How long do you take before cutting the cord?

"To be honest I can’t tell you how long I take because as I am attending to the mother, I usually don’t look at the time. But as the child gets out and I put her on the mother’s belly it’s an indicator for me to tie and cut the cord" (TBA7, Male, 34 years, Buwaiswa.

##### Child node: Antepartum Hemorrhage (APH)

The study found that TBAs recognized the dangers of APH but had limited ability to diagnose or manage it effectively. They identified APH by symptoms like vaginal bleeding and abdominal pain but couldn’t distinguish its types or risks. TBAs advised women to seek health facility care, though delays and mistrust often hindered timely referrals, leading to poor outcomes such as stillbirths and maternal complications.

*“We try our best with the few resources but as I told you early on*,* mothers trust us and we have given them medicines to heal such conditions.” (TBA15*,* Female*,* 62 years*,* Mayuge Village)*.

##### Subtheme: Admissions

TBAs acknowledged that they offered admissions to mothers who presented with some conditions like delayed contractions, birth asphyxia, etc.

*“Talking about the times you used to operate from home when they come like you’ve said maybe at night; do you provide accommodation for all of them or does the man go back and you remain with the patient only.*,* The man usually goes back home and I remain with one caretaker and the patient.” (TBA9*,* Female*,* 44 years*,* Mpungwe Village)*.

So, do you have where they sleep?

*“They just sleep in the sitting room there.”(TBA9*,* Female*,* 44 years*,* Mpungwe Village)*.

No if she has slept there is there any help you give them maybe medicine for this time or anything?

*“I give her tea and she takes because you don’t know where she has come from or whether she has eaten.”(TBA9*,* Female*,* 44 years*,* Mpungwe Village)*.

##### Subtheme: Cord around the neck

TBAs releaveled that they also manage conditions like cord around the neck using local or traditional methods.

*“Now for me in my understanding because those babies surely disturb but when the head is out try unwrapping the cord and then tell the mother to push. Because when it’s in the neck whenever she pushes and rests the baby goes back even when the head is out so whenever she pushes*,* I unwind and after a few times it releases. Because if you wait for the baby to come out it might injure it and so*,* I have a way of unwinding it. When the baby is out*,* I put her on the mother’s belly then I start tying the cord.”(TBA9*,* Female*,* 44 years*,* Mpungwe Village)*.

*“When the baby is still inside*,* I can’t tell. I just see when the baby is out.” (TBA9*,* Female*,* 44 years*,* Mpungwe Village)*.

*“You pull it out and it comes. You don’t pull it but you pull the baby if the baby has come*,* you can easily remove*,* tie and cut the cord and the baby becomes better.”(TBA9*,* Female*,* 44 years*,* Mpungwe Village)*.

##### Child node: Medicines used in managing particular conditions

TBAs use both modern medicines and traditional remedies to treat obstetric conditions. They reported using herbs, as well as pharmaceutical drugs like antibiotics, to manage infections or complications. However, TBAs noted that a lack of access to modern medical supplies sometimes hindered their ability to provide effective treatment.

*“They told us that when a mother comes when the pregnancy period is still small*,* we check them and then tell them to return after a month. If they come when the pregnancy period is advanced*,* we also tell her to return because she can come just to see whether everything is fine in the womb and when you examine her you see that everything is okay then she goes but after a short while she returns with pains.” (TBA8*,* Female*,* 38 years*,* Kikuubo Village)*.

##### Subtheme: Management of obstetric conditions

TBAs applied a variety of traditional and learned methods. One of the first steps was the identification and diagnosis of conditions. TBAs became adept at recognizing signs of preeclampsia, birth asphyxia, maternal sepsis, postdate pregnancies (Deeni), and postpartum hemorrhage. Identifying these conditions early was critical for ensuring appropriate responses. For example, it was noted that TBAs often relied on physical symptoms and community knowledge to diagnose these potentially dangerous conditions.

*“Now the mother comes with her pregnancy and requests for a checkup and I check her while putting on my gloves. Because God helped me that I saw what I was taught and still see it as if I first saw them yesterday. Now you can see the baby in the womb and how it is lying.”(TBA9*,* Female*,* 44 years*,* Mpungwe Village)*.

##### Subtheme: Specific conditions managed

TBAs had a broad scope of practice. Preeclampsia and eclampsia were managed through careful observation and, in some cases, immediate referrals to higher-level care facilities. Birth asphyxia often required quick intervention, including neonatal resuscitation techniques that TBAs were trained to administer. For conditions like postdate pregnancies (Deeni), TBAs used observation to offer all the treatments to mothers.

*“Now the mother comes with her pregnancy and requests for a checkup and I check her while putting on my gloves. Because God helped me that I saw what I was taught and still see it as if I first saw them yesterday. Now you can see the baby in the womb and how it is lying.”(TBA9*,* Female*,* 44years*,* Mpungwe Village)*.

What do you use to see? Do you use your naked eyes?

*“I use my hands to feel and know the baby’s lie and you can know the normal and abnormal lie. “Even when she comes and doesn’t know the period of pregnancy*,* you can know and tell her that “she will give birth in a certain month.”(TBA9*,* Female*,* 44 years*,* Mpungwe Village)*.

So, you only use your hands?

*“Yes*,* only in cases of listening to the baby’s heart do I use a device.” (TBA9*,* Female*,* 44 years*,* Mpungwe Village)*.

So, what do you use to listen to the fetal heart?

*“We were given some devices but mine got broken so now I don’t have.” (TBA9*,* Female*,* 44 years*,* Mpungwe Village)*.

So, for instance, today if I come and I need to know my fetal heart what do you use?

*I just do the checkup minus the fetal heart rate and that doesn’t bother me because the health Centre is nearby. And still*,* as you examine you can differentiate a live and dead baby because as you examine you feel the baby also moving in the womb.” (TBA9*,* Female*,* 44*,* years*,* Mpungwe Village)*.

Other conditions that TBAs managed included Nabuguma, a traditional term for “heat in the abdomen,” and abortions, both of which were addressed using local methods and sometimes herbal remedies. Postpartum care was another area where TBAs played a key role, offering support to mothers after childbirth to ensure recovery and address any complications. Additionally, they managed Antepartum Hemorrhage (APH), which required quick responses to prevent excessive bleeding.

*“Yes*,* I just try but it’s not ideal. I try it to stop the coldness and could be causing the shivering.”(TBA9*,* Female*,* 44 years*,* Mpungwe Village)*

Going back to “amakilo” what do you think brings them and how does paraffin help to relieve the shivering?

*“I don’t know. As I said*,* I just crammed it that way and after trying it*,* it seemed to work*,* relieving the symptoms.” (TBA9*,* Female*,* 44 years*,* Mpungwe Village)*.

*“We also use oxytocin especially when mothers take long while to get contractions and we five like one bottle on the thigh.” (TBA9*,* Female*,* 44 years*,* Mpungwe Village)*.

##### Subtheme: Infection prevention and control

Infection prevention and control were critical components of safe delivery practices. TBAs practiced various methods of waste disposal, which included traditional techniques like burial, using a placenta pit, or even disposing of waste in the garden. These practices, although not always ideal, were rooted in local customs and the resources available to TBAs. In some cases, waste was burned as a way to reduce contamination risks.

##### Subtheme: Disposal of waste

TBAs employed various waste disposal methods, including burial, burning, and using designated pits for placenta and other bio hazardous materials. They indicated that these methods were used to reduce the risk of infections, although the effectiveness of these methods varied.

*“I used to have a pit where I used to put them. When someone comes for me or comes here directly since sometimes one can just come with the mother here maybe because they don’t have another person to take care of her as he comes for me. So*,* I go and dig my pit which I know won’t be dug up by hens where I put the “kitani”.” (TBA7*,* Male*,* 46 years*,* Buwaiswa Village)*.

##### Subtheme: Personal protective equipment

The use of gloves, black plastic bags, and other personal protective equipment (PPE) was common among TBAs to reduce the risk of contamination. They reported that although PPE was useful, access to these materials was often inconsistent, which affected their ability to maintain proper hygiene.

*“You come with gloves*,* Bed sheets*,* polythene sheet razor blade*,* and the sheets for the baby.” (TBA9*,* Female*,* 44 years*,* Mpungwe Village)*.

##### Subtheme: hand washing practices

TBAs emphasized the importance of hand washing before attending to patients. They reported using soap and sometimes cooking oil to clean their hands before handling expectant mothers, which they believed helped prevent the spread of infections.

*“As you know*,* this is a village*,* a patient can come when you are just from the garden*,* and you just put on the gloves and rescue her.” (TBA7*,* Male*,* 46 years*,* Buwaiswa Village)*.

TBAs commonly used gloves and black kaveera (plastic bags) to handle contaminated materials, which helped maintain hygiene.

*“I use the gloves to examine her. Even when I am examining the abdomen alone*,* I put on gloves. So if I need to examine the other parts*,* she carries extra pairs of gloves.”(TBA1*,* Female*,* 45years*,* Buguwa Village)*.

##### Subtheme: MPDSR process

The study found that TBAs were involved in the Maternal and Perinatal Death Surveillance and Response (MPDSR) process, playing a role in reporting deaths and complications during childbirth, though their participation was often informal. They provided death notifications to local health authorities but lacked formal training to conduct detailed reviews, often relying on community leaders or health workers for further information.

*“I have never lost a mother but I have lost like two babies. The first one*,* was delivered well but after some time the baby started coughing and towards the morning it became worse so I sat on the bicycle with the baby and took him to the Buwaiswa*,* on reaching he was dead. And the other one; the mother came here while in labor*,* on giving birth the baby’s head had no bone so I think the baby was born dead. Otherwise*,* I have not lost any other babies.”(TBA3*,* Female*,* 52 years*,* Nawanvubu Village)*.

##### Subtheme: Death notifications

The study found that TBAs were involved in the Maternal and Perinatal Death Surveillance and Response (MPDSR) process, playing a role in reporting deaths and complications during childbirth, though their participation was often informal. They provided death notifications to local health authorities but lacked formal training to conduct detailed reviews, often relying on community leaders or health workers for further information.

*“I have never lost a mother but I have lost like two babies but I only reported to the relatives and told them to pick their dead bodies.”(TBA15*,* Female*,* 53 years*,* Mayuge Village)*.

##### Subtheme: Perinatal death reviews

The study found that TBAs were involved in the Maternal and Perinatal Death Surveillance and Response (MPDSR) process, playing a role in reporting deaths and complications during childbirth, though their participation was often informal. They provided death notifications to local health authorities but lacked formal training to conduct detailed reviews, often relying on community leaders or health workers for further information.

*“We don’t review anything*,* those days when we were working peacefully*,* we would report to the nearby health facility and they review but they don’t even tell us the cause of death.”(TBA12*,* Female*,* 39 years*,* Nansaga Village)*.

##### Subtheme: Maternal death reviews

Similarly, TBAs played a limited role in maternal death reviews, though they were sometimes included in community discussions about the causes. In rural areas, they often knew the circumstances of the deaths they attended, but lacked access to formal data or resources for systematic reviews. TBAs expressed a desire for more training and involvement in the process. The study emphasized the importance of including TBAs more actively, as they were often the first point of contact and could provide valuable insights into maternal fatalities.

*“Yes*,* before we stopped actively working*,* we used to even make monthly reports on the people we have helped*,* the number of babies*,* how many are alive and how many are dead plus any complications in all the cases.”(TBA11*,* Female*,* 38years*,* Mayuge Village)*.

##### Subtheme: Referral process

The study found that TBAs understood the importance of referrals, especially during complications beyond their capacity. However, they faced challenges such as poor transport, long distances, and family reluctance to seek formal care. In some cases, they referred clients to other TBAs or traditional healers based on the situation and family preferences.

##### Child node: Referral to the health facility

The study found that TBAs often referred women to health facilities during emergencies like hemorrhage, preeclampsia, or obstructed labor. Although they recognized the need for medical care, referrals were hindered by transport issues, patient reluctance, and a lack of clear referral protocols, leaving TBAs unsure about proper procedures.

No if the mother is okay, I don’t go with her. The only one I go with is the one who comes in a bad condition, the one that I believe I cannot handle. I go with her because I can’t help her.” (TBA3, Female, 53 years, Nawanvubu Village).

To the hospital, even if they reach at what time in the night, I discharge them, maybe if they came in at that time when I haven’t seen them and recognize them.” (TBA7 Male, 46years, Buwaiswa Village).

##### Subtheme: TBA to TBA referral

When unable to manage complications, TBAs often referred women to more experienced TBAs within their community, especially where healthcare access was limited. This informal TBA-to-TBA referral system provided a safety net but had limitations. Due to varied training and practices, care quality differed, and referring TBAs were sometimes uncertain if the receiving TBA had the expertise to handle the complication.

*“There some people who might come saying they went to the scan and were told the baby is not lying well and you check and find out its true so I keep checking her and after some time*,* if she came early you see it has changed Position so you can send her for a scan and confirm but if you check and it’s still not in the right position you tell her to go to one of the senior TBAs in Bugweri district.”(TBA13*,* Female*,* 43 years*,* Bukatuube Village)*.

##### Subtheme: Referral to traditional healers

The study found that TBAs sometimes referred women to traditional healers for conditions believed to have cultural or spiritual causes, such as Nabuguma (heat in the uterus). These referrals occurred when TBAs felt unable to treat the condition or when patients preferred traditional care. While culturally accepted, this led to overlapping treatments and conflicting advice, which negatively affected patient outcomes.

*“We refer to the traditional healers especially those with Amakiro*,* they calm back when they are very fine.”(TBA9*,* Female*,* 44 years*,* Mpungwe Village)*.

*“There are conditions which we can’t manage for example when a mother reaches 40 weeks but the baby can’t move*,* we refer them for spiritual intervention.” (TBA15*,* Female*,* 62 years*,* Mayuge Village)*.

##### Theme: Roles and influences of social-cultural-economic factors

Social and cultural factors greatly influenced TBAs’ practices and effectiveness. Poverty often forced families to rely on TBAs due to the high cost of formal care. Husbands’ preferences for traditional births or fear of medical facilities also affected care choices. Community support shaped how TBAs operated and were perceived. Many TBAs were motivated by a strong sense of duty and were supported by local leaders and the community who valued their services.

##### Child node: Poverty among mothers

Economic constraints were found to influence the decision to use TBA services by mothers. TBAs reported that many mothers could not afford the costs associated with hospital-based care, leading them to seek out TBAs for assistance with childbirth.

It is just that sometimes she might come in pain and tell you that she lacks transport to the facility and her husband is not around so she asks you that in case the time comes you assist her but if the husband comes back then they might go to the facility. Sometimes it is really night and nowadays there are a lot of killings, they fear that they might be attacked on the way to hospital. Or sometimes they might come for you here to go and assist them from their home because right now I don’t have where they can give birth from.”(TBA6, Female, 60years, Kigulu Village.

##### Child node: Husband influence

Husbands played a significant role in deciding where a woman would deliver. TBAs shared that some husbands preferred the services of TBAs, often due to cultural beliefs or fear of hospital delivery, which influenced maternal care choices.

*“Some mothers are brought here by their husbands to receive services and they even tell us to keep giving them feedback. Even if the situation gets worse*,* they still tell us to treat me.”(TBA5 Female*,* 54 years*,* Buwalima Village)*.

*“We also deliver wives of our fellow male TBAs (name mentioned) because they can’t deliver them so they refer them to us.”(TBA10*,* Female*,* 32 years*,* Bukatuube Village)*.

##### Child node: Motivation by community

The community’s trust and reliance on TBAs motivated them to continue their work despite the challenges. TBAs reported that the sense of duty to the community, along with recognition of their role in maternal health, kept them engaged in providing care.

*“Some people prefer us to health workers and they have supported our existence*,* actually when the government started fighting us*,* some mothers stopped going to facilities in protest*,* and that time I had the highest number of deliveries*,* over 6 per day.”(TBA13*,* Female*,* 33 years*,* Bukatuube Village)*.

##### Child node: Customer care at TBA sites

Traditional Birth attendants offered customer care services to their clients which included a cup of tea and lunch as well as other support like washing for them after delivery.

*“No may be giving her some tea. Because some people come in labor and have not had breakfast lunch or supper and sometimes*,* I even give them some food if I have to help them regain their body strength?”(TBA6*,* Female*,* 60 years*,* Kigulu Village)*.

#### Theme: Challenges faced by TBAs in providing safe and effective maternal and child health care services

TBAs have faced numerous challenges, including official bans from health authorities and competition from both other TBAs and private clinics. They worked under fear of sanctions, lacked support from the Ministry of Health, and struggled with resource limitations such as inadequate supplies like gloves and plastic bags. Additionally, patient negligence, such as delays in seeking care and poor antenatal care attendance, had complicated their ability to provide safe and effective services. Despite these challenges, TBAs have remained vital in areas where formal healthcare services were scarce.

##### Subtheme: Ban by the ministry of health

Many TBAs operated under fear of sanctions or arrest, which stifled their ability to offer services freely. Additionally, competition among TBAs was common, both in terms of reputation and resources. The lack of support from the MOH compounded these challenges, as TBAs often lacked access to official resources and formal recognition.

*“I think the government should be open and accept us to work because we are also people. They should allow us to work as we used to. If they can also get us somewhere*,* where we can take mothers who have complications and maybe improve on transportation of patients here.”(TBA11*,* Female*,* 38 years*,* Mayuge Village)*.

##### Subtheme: The lack of sundries by patients

Many patients could not afford basic supplies such as gloves or plastic bags, which made it difficult for TBAs to maintain proper hygiene.

*“Some patients come here with nothing*,* you are forced to get your own clothes like gomesi tear and give them to use for taking the baby home.”(TBA2*,* Female*,* 34 years*,* Wangobo Village)*.

*“There are patients who come with one pair yet they are to be admitted for long which makes us end up reusing them*,* that I mean we wash them and keep using them.” (TBA1*,* Female*,* 45 years*,* Buguwa Village)*.

##### Subtheme: Negligence of patients

It was reported that many patients delayed seeking care or did not follow appropriate treatment protocols. The delay to reach the TBA facility was a common issue, with women often waiting too long to seek help during labor. Poor antenatal care (ANC) visits further compounded the problem, as many women did not attend regular checkups, leading to undiagnosed complications and increased risks.

*“There are these mothers who first try to deliver by themselves at home and come here when the kitani (placenta) is already out*,* they over bleed which makes our work hard.” (TBA9*,* Female*,* 44years*,* Mpungwe Village)*.

##### Subtheme: working under fear

The study found that many TBAs lived in constant fear of Ministry of Health restrictions on untrained birth attendants. They worried about legal penalties and often worked secretly to avoid being reported. This fear limited their confidence and scope of care. Despite this, TBAs felt compelled to continue due to the urgent need for maternal care in rural areas with limited formal healthcare access.

*“When they started talking about us on radios and telling us ‘don’t do this*,* don’t do that’ I decided that if someone wants my services*,* I gave them a rule that they just call me and I go to their place and I assist them from there. Because me here I no longer have the room. I removed it. But those days I used to admit when I still had the room.”(TBA7*,* Female*,* 46years*,* Buwaiswa Village)*.

##### Subtheme: Lack of support from the ministry of health

The study revealed that many TBAs felt neglected by the MOH, citing inconsistent training, limited resources, and lack of recognition. While some training existed, it was often infrequent and inaccessible. TBAs felt viewed as informal providers, receiving little guidance or support, forcing them to work independently and affecting care quality. They suggested that better collaboration and support from the MOH could improve maternal healthcare in their communities.

*“I think the ministry should just support us and not fight us by the way*,* can they train us and take us to health facilities or even work with us. Their lack of support has made us lose morale of working.” (TBA9*,* Female*,* 44 years*,* Mpungwe Village)*

Ministry of Health has not supported us instead they have tortured us yet we are the people who delivered their grandmothers etc., she cries…… (TBA15, Female, 60years, Mayuge Village).

##### Subtheme: Threats from district leadership

The study highlighted that TBAs faced threats from district leadership aligned with MOH policies, including fines, arrests, and business shutdowns for providing services. This created constant anxiety and reluctance among TBAs to seek official support, as they feared punishment rather than assistance. The tension stemmed from policies aiming to exclude TBAs from maternal healthcare.

*“There is a team of people from the district which keeps coming and tells us that we are going to be arrested*,* we end up working while hiding.”(TBA6*,* Female*,* 60 years*,* Kigulu Village)*.

##### Subtheme: TBA vs. TBA competition

The study revealed that competition among TBAs was a challenge in some regions, leading to rivalries for clients. This sometimes reduced care quality, as TBAs felt pressured to offer faster or cheaper services. Competition also hindered collaboration and knowledge sharing, causing distrust within communities. Despite this, many TBAs valued peer relationships and found ways to cooperate, recognizing their joint role in improving maternal health.

*“You are aware*,* we are many in Mayuge and everyone is looking for survival so some of our colleagues who have money employ midwives to keep referring mothers to them.” (TBA13*,* Female*,* 42 years*,* Bukatuube Village)*.

*“Some of the traditional birth attendants have agents with in the community*,* so find them at bore holes*,* community SACCO meetings encouraging mothers to go to that particular TBA*,* so us who don’t have*,* we don’t get them.” (TBA4*,* Female*,* 38 years*,* Baitambogwe)*.

##### Subtheme: Competition with private clinics

The study found that competition from private clinics posed a challenge for TBAs, as many women preferred the advanced care, better facilities, and specialized staff offered by these clinics. This shift left TBAs feeling marginalized and undervalued, unable to compete with formal healthcare resources and training. However, private clinics were often too costly for women in rural or poor areas, so TBAs remained essential, especially in remote regions where clinics were scarce or absent.

*“There are private clinics in Mayuge*,* they discourage mothers from coming to us but they later come after being mismanaged.”(TBA10*,* Female*,* 32 years*,* Bukatuube Village)*.

##### Subtheme: Not taking appropriate treatment

The study revealed that many women did not follow treatment or advice from TBAs, often due to cultural beliefs, mistrust of formal healthcare, or family preference for traditional healing. Some neglected essential postnatal care, which frustrated TBAs who believed proper follow-up could prevent many complications.

*“You give a mother treatment to take like 4 times a day but because she delayed in the garden*,* she ends up taking only once*,* which is not right.” (TBA12*,* Female*,* 39 years*,* Nansaga Village)*.

*“Then there are these young mothers who come here for local herbs like to stop vomiting during pregnancy*,* they mix what they are given at the facility and what we give them which exacerbates ore problems.”(TBA5*,* Female*,* 54 years*,* Buwalima Village)*.

##### Subtheme: Poor Antenatal Care (ANC) visits

The study highlighted poor ANC attendance as a common issue. TBAs reported that many women missed visits due to lack of awareness, financial constraints, or fear of medical settings. This led to TBAs managing high-risk pregnancies that regular prenatal care could have better addressed. Participants stressed that inadequate prenatal care contributed to preventable complications like preeclampsia and postpartum hemorrhage and called for more community education on the importance of ANC and early intervention.

*“Now there is a big challenge her*,* mothers come here for a full package of ANC then they also go to Mayuge HCIV*,* they mix the two end up getting problems*,* I wonder why they can’t do one at ago.” (TBA8*,* Female*,* 39 years*,* Kikuubo Village)*.

#### Theme: Contextual factors

TBAs operated in environments with various cultural, social, and economic challenges. In some communities, traditional beliefs discouraged women from seeking modern medical care, making it difficult for TBAs to encourage hospital deliveries. Additionally, poverty limited access to essential resources, such as clean delivery kits, making safe deliveries more challenging.

##### Subtheme: Failure of mothers to disclose their HIV status

Many expectant mothers did not disclose their HIV status to TBAs due to fear of stigma or lack of awareness. This put both the TBA and the newborn at risk of transmission during delivery. Without this crucial information, TBAs were unable to take necessary precautions or provide appropriate interventions to protect both the mother and child.

*“Women don’t want to tell us their HIV status especially when they come with their husbands which makes our work hard.” (TBA4*,* Female*,* 38 years*,* Waitambobwe)*.

##### Subtheme: Lack of HIV testing kits

TBAs often lacked access to HIV testing kits, making it difficult to identify HIV-positive mothers. Early detection is crucial in preventing mother-to-child transmission through interventions like antiretroviral therapy and safe delivery practices. Without testing kits, TBAs were unable to make informed decisions regarding the management of such cases.

*“We can’t keep testing every woman who comes because we can’t access enough kits*,* especially seasLns when deliveries are many.” (TBA12*,* Female*,* 39 years*,* Nansaga Village)*.

##### Subtheme: Lack of transport to health facilities for complicated cases

In cases of complications like prolonged labor, excessive bleeding, or fetal distress, timely referral to a health facility was critical however, many TBAs operated in remote areas with poor transport infrastructure. The lack of ambulances or any means of transport put the lives of both the mother and baby at great risk.

*“In case of complicated cases*,* I just call the boda boda man and they take to Iganga or Mayuge but I have no ambulance ……she laughs.” (TBA7*,* Male*,* 46 years*,* Buwaiswa Village)*.

#### Theme: Harsh attitude from health workers

When TBAs referred complicated cases to health facilities, they often faced hostility from healthcare workers. Instead of collaborating with TBAs to ensure safe deliveries, some health workers dismissed or criticized them, discouraging them from making referrals in the future. This attitude created a gap between traditional and modern healthcare, ultimately affecting maternal and child health outcomes.

*“You don’t know how those midwives …. Mention a name…. treat us when we refer mothers there*,* others frit asks for money before attending to pour patients.” (TBA15*,* Female*,* 62 years*,* Mayuge Village)*.

#### Theme: Lack of space for deliveries and postnatal care

Many TBAs operated from their homes or makeshift facilities without proper delivery rooms or resting spaces for mothers after childbirth. The absence of hygienic and adequately equipped spaces increased the risk of infections, postpartum complications, and poor recovery for new mothers.

*“I sometimes deliver my mother’s from my neighbor’s place*,* just like you see*,* the rain disorganized my house and delivery room as well.” (TBA9*,* Female*,* 44 years*,* Mpungwe Village)*.

##### Subtheme: Unprepared mothers for delivery

Some pregnant women sought TBAs’ services without adequate preparation for childbirth, lacking essential supplies like clean clothes, sanitary pads, or even funds for emergency transport. This lack of preparedness increased the risk of complications and limits the ability of TBAs to provide safe and effective care.

*“There are some mothers who walk in here as if they have come to have lunch or even greet you*,* they end up having lunch with us then all of a sudden they start getting contractions*,* they have not come with anything and I end up giving them my gomesis.” (TBA12*,* Female*,* 39 years*,* Nansaga Village)*.

## Discussion

This study sought to explore the evolution, experiences, practices, challenges, and perceived support needs of Traditional Birth Attendants (TBAs) in Mayuge District, Uganda, and examine their interaction with formal health structures. The findings provide nuanced insights into the multifaceted role of TBAs within their communities, highlighting their diverse pathways into practice, the hybrid nature of their skills, their adaptive approaches to care, and the complex socio-cultural and systemic challenges they face.

TBAs in Mayuge entered the practice through multiple pathways including informal apprenticeships, formal trainings by the Ministry of Health (MOH) or NGOs, and transitions from Village Health Teams (VHTs). Many TBAs described learning through hands-on mentoring by experienced relatives, with statements such as, “I followed my grandmother during deliveries until I could handle alone,” reflecting the strong oral and practical tradition underpinning TBA knowledge. Others reported attending MOH-led or NGO workshops that provided structured content such as antenatal care and newborn resuscitation. The transition of some TBAs from VHT roles to birth attendants illustrates a fluidity within community health roles, driven by local demand and perceptions of the formal referral system’s limitations. Similar patterns have been documented in Nigeria [10], where TBAs often emerge from other community health roles to fill service gaps in maternal care. This blend of informal and formal learning resulted in heterogeneous skill levels among TBAs, a finding consistent with research in Tanzania where TBAs’ competencies varied according to their training histories [4]. Such diversity has important implications for programmatic engagement, emphasizing the need for tailored support that respects varied learning backgrounds.

The TBAs exhibited a considerable ability to recognize signs of maternal and neonatal complications including preeclampsia, postpartum hemorrhage, breech presentations, and neonatal asphyxia. Many described clinical signs accurately, such as swollen bodies and headaches indicative of preeclampsia, and reported employing both traditional remedies and basic biomedical interventions. For example, herbal preparations were commonly used to stimulate labor or alleviate abdominal discomfort (referred to locally as nabuguma), alongside referral to health facilities for complicated cases. This integration of traditional and biomedical practices echoes findings from Ethiopia, where TBAs combine indigenous knowledge with modern practices to adapt to resource constraints [11]. The pragmatic use of both traditional herbs and limited modern medicines by TBAs in Mayuge reflects their role as frontline responders in contexts where formal services are distant or inaccessible [11]. However, this hybrid approach also raises challenges for standardizing care quality and safety, an issue noted in Kenya where variations in herbal use led to inconsistent outcomes [12].

TBAs demonstrated awareness of infection prevention practices such as handwashing with soap, use of gloves, and safe disposal of delivery waste by burial or burning. However, chronic shortages of supplies led to improvised methods including the use of polythene bags (*kaveera*) as protective barriers and substitution of cooking oil for soap during hand washing. One TBA explained, “If a mother comes without gloves, I use my own. If I have none, I use a plastic bag.” These findings highlight a critical tension: while TBAs understand IPC principles, systemic neglect and supply chain failures force them into suboptimal protective practices [13]. Similar observations were reported in Malawi, where TBAs’ IPC adherence was limited by lack of gloves and clean water [14]. Such local adaptations, although ingenious, may increase risk of infection transmission, underscoring the need for targeted support that supplies basic protective materials and builds on existing IPC knowledge [15].

Despite their unofficial status, TBAs in Mayuge participate in Maternal and Perinatal Death Surveillance and Response (MPDSR) through death notifications and occasional participation in review meetings [16]. They also actively refer complicated cases to health facilities or more skilled TBAs, and sometimes to traditional healers when spiritual causes are suspected [17]. The referral decisions are influenced by multiple factors including recognition of their own limits [18], cultural beliefs [19], transport availability, and experiences of negative attitudes at health centers. This nuanced referral behavior aligns with findings from Nepal, where TBAs act as key intermediaries linking communities with formal services but face barriers such as disrespectful treatment and transport difficulties [20]. The pluralistic health environment described by Mayuge TBAs, combining biomedical, traditional, and spiritual approaches, mirrors patterns in Ghana where care-seeking pathways are fluid and complex [7].

Cultural expectations, particularly the influence of husbands and mothers-in-law, significantly shaped delivery location and care choices. One TBA recounted, “The man said, ‘Don’t take my wife to the hospital. She will die there,’” highlighting fears and misconceptions that limit facility births. Poverty compounded these barriers, with many mothers unable to afford transport or delivery supplies such as gloves, leading to unsafe delivery environments [21]. This social contract, in which TBAs provide care irrespective of payment, is central to their sustained relevance. As one TBA noted, “Even when they have nothing to pay, I help them. That’s why they keep coming.” Similar socio-economic and gender dynamics influencing maternal health decisions have been extensively reported in Uganda and elsewhere [2, 22]. These findings stress the importance of addressing both financial and gendered power structures to improve maternal health outcomes.

TBAs operate under the shadow of the Ministry of Health’s ban on TBA-assisted deliveries, forcing them into secrecy and limiting open collaboration or learning opportunities [15]. Many expressed fear of arrest, feeling criminalized despite their vital role, with one stating, “We are afraid to be arrested, yet we are the ones helping mothers here.” This precarious legal environment undermines referral transparency and exacerbates mistrust with formal providers [23].

Additional challenges include lack of HIV testing kits, transport shortages, harsh attitudes from health workers, and competition from private clinics and other community health actors [24]. The absence of MOH recognition and supplies “They don’t even give us gloves” left many TBAs feeling undervalued despite their frontline contributions. Rivalries among TBAs and with VHTs further fractured community health efforts [25].

Such systemic neglect and stigmatization mirror experiences reported in Sierra Leone and Malawi, where TBAs operate in legal and policy limbo with limited institutional support [26]. These challenges highlight the need for pragmatic policies that acknowledge TBAs’ de facto roles and seek collaborative rather than punitive approaches [27].

TBAs described their relationships with formal health systems as distant and sometimes adversarial. While some had previously engaged in MOH or NGO trainings, most felt excluded from ongoing initiatives. Referral processes were often marked by disrespect, lack of feedback, and institutional rigidity; as one respondent lamented, “When we bring a mother, they don’t even say thank you.” Despite this, several TBAs expressed a willingness to collaborate if their roles were recognized and respected. Their informal contributions to MPDSR and referral networks demonstrate an existing but fragile integration into maternal health systems [5]. This liminal position both essential and disallowed reflects findings from Ethiopia and Tanzania, where TBAs operate in parallel with, but largely outside of, formal health structures [28]. This complexity underscores the need for policy approaches that are pragmatic, culturally sensitive, and inclusive of TBAs’ lived realities. Rather than aiming for wholesale replacement, health systems should consider ways to engage and support TBAs as community allies in improving maternal and newborn health outcomes.

## Conclusion

The findings of this study illuminate the complex, evolving role of Traditional Birth Attendants in Mayuge District, Uganda. Rooted in a blend of apprenticeship, formal training, and community recognition, TBAs embody a hybrid knowledge system that combines traditional practices with selected biomedical skills. They play an indispensable role in identifying and managing maternal and neonatal complications, often under conditions constrained by limited resources and systemic neglect. Despite operating under legal restrictions and facing stigma, TBAs continue to provide essential services driven by deep community trust and socio-cultural obligations. Their pragmatic infection prevention practices, participation in death surveillance, and referral efforts highlight their potential as critical allies in improving maternal and newborn health outcomes. However, persistent challenges including lack of formal recognition, inadequate supplies, negative attitudes from health workers, and economic and cultural barriers limit their effectiveness and integration within the formal health system. Addressing these gaps requires pragmatic, inclusive policies that acknowledge TBAs’ lived realities and leverage their unique position in communities. In conclusion, rather than marginalizing TBAs, maternal health strategies in Uganda and similar settings should focus on collaborative engagement, capacity building, and respectful integration to optimize outcomes for mothers and newborns in underserved areas.

## Supplementary Information

Supplementary Material 1.

Supplementary Material 2.

## Data Availability

The transcripts used and/or analyzed during the current study are available from the corresponding author upon reasonable request.
