# Supplementary material for: Catching babies, carrying traditions: the voices and practices of traditional birth attendants in Mayuge District, East central Uganda
Source: Reprod Health. 2026 Jan 10;23:40. doi: 10.1186/s12978-025-02251-3 (PMC12882226; doi:10.1186/s12978-025-02251-3)
Supplement: Supplementary file 1 — Supplementary Material 1. [file 12978_2025_2251_MOESM1_ESM.docx]

April, 2025


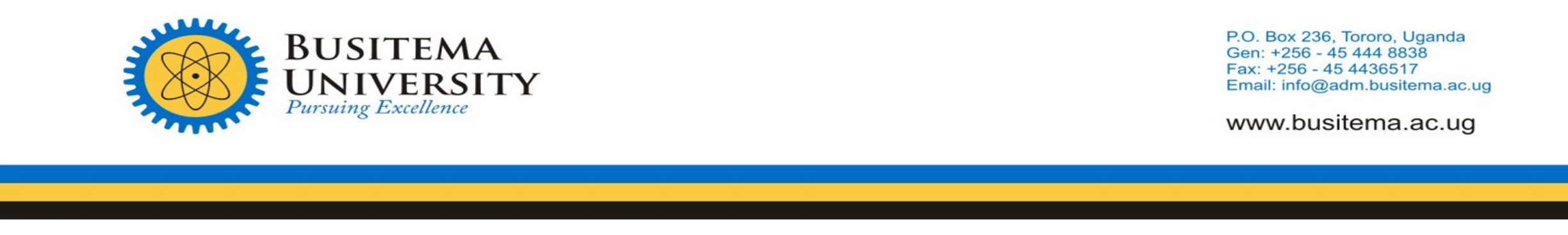


**The Editor**
BMC Pregnancy and Childbirth

Dear Sir/Madam,

**RE: Manuscript Submission “Catching Babies, Carrying Traditions: The Voices and Practices of Traditional Birth Attendants in Mayuge District, East Central Uganda”**

On behalf of the authorship team, I am pleased to submit our manuscript titled “Catching Babies, Carrying Traditions: The Voices and Practices of Traditional Birth Attendants in Mayuge District, East Central Uganda” for your kind consideration for peer review and potential publication in BMC Pregnancy and Childbirth.

This study presents novel and significant findings from research conducted in the Busoga region of Uganda. It explores the roles of Traditional Birth Attendants (TBAs), assesses their practices and training needs, and critically evaluates the current policy frameworks intended to integrate TBAs into formal healthcare systems. The findings are highly relevant and timely, providing important insights that can inform the Ministry of Health and other stakeholders in developing effective strategies to enhance maternal and child health services in rural Eastern Uganda.

Given the urgency and importance of the topic, the study was conducted with limited financial support, primarily allocated to facilitate data collection. In light of this, we respectfully request a waiver for the Article Processing Charges to enable the publication and dissemination of these impactful findings. We believe this contribution will significantly enrich the existing body of scientific knowledge in maternal and child health.

We sincerely thank you for considering our manuscript and our waiver request.

Yours faithfully,
**Enid Kawala Kagoya**
Corresponding Author
