# Supplementary material for: Catching babies, carrying traditions: the voices and practices of traditional birth attendants in Mayuge District, East central Uganda
Source: Reprod Health. 2026 Jan 10;23:40. doi: 10.1186/s12978-025-02251-3 (PMC12882226; doi:10.1186/s12978-025-02251-3)
Supplement: Supplementary file 2 — Supplementary Material 2. [file 12978_2025_2251_MOESM2_ESM.pdf]

Semi-Structured Interview Guide: Traditional Birth Attendants (TBAs) in Mayuge District

Introductory Script:

Thank you for agreeing to this interview. We are interested in learning about your work, experiences, and challenges as a Traditional Birth Attendant. There are no right or wrong answers we want to hear your stories and views. Your responses will be kept confidential and used only for research purposes.

Main Question:

- Can you tell me how you became a TBA?

Prompts/Probes:

- Did someone teach or mentor you? Who?
- Did you receive any training from the Ministry of Health or NGOs?
- Were you ever a VHT? How did that relate to your work as a TBA?
- What made you decide to become a TBA?
- How long have you been practicing?

Main Question:

- What types of care do you provide to mothers and babies?

Prompts/Probes:

- Can you describe what you do during pregnancy, delivery, and after birth?
- What methods or tools do you use (e.g., herbs, medicines, gloves)?
- How do you recognize danger signs like heavy bleeding or breech babies? Observe
- How do you manage complications such as a baby not breathing at birth, or seizures in the mother? Observe
- Do you ever use local herbs or modern drugs? In what situations?

Main Question:

- How do you keep mothers, babies, and yourself safe from infection during delivery? Observe …

Prompts/Probes:

- Do you wash hands before and after deliveries? What do you use? Observe ….
- Do you use gloves or other protective materials? How do you get them? Ask for them
- How do you dispose of items like placenta, gloves, or bloody cloths? Observe..
- What challenges do you face in following safe hygiene practices?

Main Question:

- What happens when a mother or baby dies under your care or in your community?

Prompts/Probes:

- Do you report the death? To whom?
- Have you ever been invited to discuss the cause of death?
- What do you think should happen after such a death?
- What role do you feel TBAs should have in preventing or responding to these deaths?

Main Question:

- Can you describe what you do when a mother needs more help than you can provide?

Prompts/Probes:

- When do you refer a mother to a health facility?
- How do you decide where to refer her?
- Do you sometimes refer to another TBA or a traditional healer?
- What difficulties do you face when referring (e.g., transport, communication, reception at the facility)?
- How do health workers respond when you refer a mother?

Main Question:

- How does the community influence your work as a TBA?

Prompts/Probes:

- Why do some women prefer TBAs over health facilities?
- How do money, distance, or family roles (husbands, mother-in-laws) affect care decisions?
- Are there cultural beliefs or taboos that guide how you do your work?
- How are you viewed in your community? Respected, supported, or criticized?
- Are you treated differently when working with different tribes or religious groups?

Main Question:

- What challenges do you face in your work?

Prompts/Probes:

- Do you have the materials and medicines you need?
- Are there government restrictions on your work?
- What do you do when you suspect a mother has HIV? Do you test or refer?
- How do you handle late arrivals or mothers with no supplies?
- Are there disagreements with health workers, VHTs, or other TBAs?
- What support or recognition do you get from health facilities or the government?

Closing Questions

Main Question:

- What else would you like to share about your work?

Prompts/Probes:

- What support would help you serve mothers better?
- How do you see your role changing in the future
